# Is assisted oocyte activation an effective method in poor responders?

**DOI:** 10.1007/s00404-026-08491-3

**Published:** 2026-07-16

**Authors:** Ayşe Altun Akpinar, Burçin Karamustafaoğlu Balcı, Çiğdem Çelik, Cemil Akgül

**Affiliations:** Department of Obstetrics and Gynecology, Istanbul Faculty of Medicine, Istanbul University, Istanbul, Turkey

**Keywords:** Chemical activation, Electrical activation, Fertilization, Poor responders

## Abstract

**Objective:**

Poor responders pose a significant challenge in fertility treatment, leading to decreased oocyte quality and fertilization rates. Assisted oocyte activation (AOA) has emerged as a method to improve fertilization rates. This study aims to evaluate the effect of AOA on fertilization, pregnancy, and live birth rates in patients with diminished ovarian reserve.

**Materials and methods:**

This prospective non-randomized comparative study included a cohort of infertility patients with diminished ovarian reserve, divided into three groups: the control group receiving standard treatment (Group I/Conventional ICSI), the electrical activation group undergoing piezoelectric stimulation (Group II/ICSI + Piezoelectric), and the chemical activation group treated with calcium ionophore (Group III/ICSI + Calcium Ionophore). Each group consisted of 50 patients. The effects of electrical and chemical activation applied after ICSI on fertilization, pregnancy, and live birth rates were evaluated.

**Results:**

There were no statistically significant differences among the groups in hormone levels (FSH, LH, E2, PRL, and AMH), number of oocytes, or number of MII oocytes. Fertilization rates were significantly higher in the piezoelectric group compared to the control group (*p* = 0.007). Although the fertilization rate in the calcium ionophore group was higher than that in the control group, this difference did not reach statistical significance (*p* = 1.000). There was no statistically significant difference in fertilization rates between the piezoelectric and calcium ionophore groups (*p* = 0.071). Regarding pregnancy rates, the highest implantation rate was observed in the calcium ionophore group (37%), but this difference does not reach statistical significance (*p* = 0.224). No significant differences were observed among the groups in embryo quality, clinical pregnancy, ongoing pregnancy, and live birth rates.

**Conclusion:**

In patients classified as diminished ovarian reserve, artificial oocyte activation (AOA) was associated with higher fertilization rates for increasing fertilization rates, potentially resulting in a greater number of fertilized oocytes and transferable embryos. However, this apparent benefit does not translate into improved biochemical pregnancy outcomes. Moreover, no significant differences were observed between groups in terms of embryo quality, clinical pregnancy, ongoing pregnancy, and live birth rates. These findings suggest that while AOA may enhance early laboratory outcomes, its impact on clinically meaningful reproductive endpoints remains limited. Therefore, further adequately powered randomized studies are required to clarify the efficacy and safety of AOA.

## What does this study add to the clinical work?

**Table:** 

Artificial oocyte activation may improve early laboratory outcomes in patients with diminished ovarian reserve, particularly fertilization rates after piezoelectric activation. However, this improvement did not translate into significantly better embryo quality, clinical pregnancy, ongoing pregnancy, or live birth rates. Therefore, AOA should be considered an individualized adjunct rather than a routine intervention in poor responders until adequately powered randomized studiesconfirm its clinical benefit.	

## Introduction

Diminished ovarian reserve (DOR) is defined as a reduction in both the number of follicles and/or the quality of oocytes in a woman’s ovaries [1]. It is one of the leading causes of infertility, particularly in women of advanced age. Non-age-related DOR may result from oxidative stress, genetic factors, early follicular atresia, or exposure to environmental toxins.

Patients with DOR constitute the majority of those who exhibit poor responses to ovarian stimulation in IVF cycles and are associated with high cycle cancelation rates, as well as low fertilization and pregnancy rates [2, 3]. Numerous techniques have been explored to improve fertilization rates, offering hope for couples affected by DOR. Consequently, artificial activation techniques now provide an alternative to conventional IVF approaches for these patients.

Artificial oocyte activation (AOA) methods are either chemical or mechanical, with calcium ionophore being one of the chemical methods. For oocyte activation to occur, the sperm must first complete capacitation. Subsequently, the release of Ca^2^⁺ ions is required to trigger sperm hyperactivation and the acrosome reaction [4]. Oocyte activation is believed to depend on calcium (Ca^2^⁺) release [5]. Calcium ionophores facilitate the passage of calcium ions across the cell membrane, increasing intracellular calcium concentration, thereby promoting oocyte activation and contributing to fertilization. Several studies have shown that calcium ionophore use can improve fertilization rates, clinical pregnancy rates, and live birth outcomes [6].

The piezoelectric-assisted oocyte activation is a type of mechanical activation method that enables the passage of calcium ions (Ca^2^⁺) into the cell by delivering electrical stimulation at a specific current intensity and duration. The electrical signal stimulates ion channels in the oocyte membrane and cytoplasm, triggering a rapid and controlled influx of calcium ions into the oocyte. This intracellular increase in Ca^2^⁺ initiates fertilization mechanisms and is considered the most critical signal for oocyte activation. The piezoelectric-assisted oocyte activation allows oocyte activation to be achieved through an electrical rather than a chemical method. Studies suggest that this approach is particularly effective in increasing fertilization rates and improving embryo quality in cases of total fertilization failure and severe sperm abnormalities [7].

Although conventional IVF remains the first-line fertilization method in the absence of male factor infertility, ICSI is frequently preferred in patients with diminished ovarian reserve to reduce the risk of fertilization failure when only a limited number of oocytes are obtained. In cases with a good ovarian response, when numerous MII oocytes are available, the embryologist may choose conventional intracytoplasmic sperm injection (ICSI) or, if necessary, any of the artificial oocyte activation methods. The oocytes may even be split to attempt both approaches. However, when only a few high-quality MII oocytes are present, the optimal strategy—conventional ICSI, AOA with calcium ionophores, or AOA with the piezoelectric-assisted oocyte activation—remains unclear. This study aims to compare these three methods in poor responders and to determine the most effective approach for achieving improved IVF outcomes. Although current guidelines primarily recommend assisted oocyte activation for specific indications such as total fertilization failure or severe male factor infertility, the present study explores the potential role of AOA in a highly selected group of patients with diminished ovarian reserve**,** in whom only a very limited number of oocytes can be obtained and each IVF cycle carries substantial clinical and emotional burden.

## Materials and methods

This prospective non-randomized comparative study was conducted between October 2023 and May 2025 in the Department of Reproductive Endocrinology and Infertility at Istanbul Faculty of Medicine, Istanbul University. This prospective non-randomized comparative study was designed to evaluate embryological and clinical outcomes following different assisted oocyte activation strategies. The primary outcome of this study was the fertilization rate (2PN) following intracytoplasmic sperm injection with or without assisted oocyte activation. Secondary outcomes included embryo developmental quality, implantation rate, clinical pregnancy rate, ongoing pregnancy, and live birth rate.

The study population consisted of women with diminished ovarian reserve undergoing IVF/ICSI treatment at our center, with “poor responders” defined according to the Bologna criteria [8].

Patients aged 18–40 years with diminished ovarian reserve were included if they were nulliparous and underwent IVF/ICSI cycles using fresh ejaculated sperm with normozoospermic parameters according to WHO criteria. Diminished ovarian reserve was defined as an anti-Müllerian hormone (AMH) level < 1.1 ng/mL measured within six months prior to treatment.

Patients were excluded if they had a history of previous pregnancy or live birth, endometriosis, age > 40 years, or normal ovarian reserve. Additional exclusion criteria included abnormal sperm parameters, the use of frozen–thawed sperm, uterine pathologies affecting implantation (including hydrosalpinx, uterine septum, intrauterine polyps, or submucosal fibroids), and severe systemic or endocrine disorders that could influence reproductive outcomes.

During the study period, patients who fulfilled the inclusion and exclusion criteria and consented to participate were included in the study. To minimize potential confounding effects and to maintain a focused evaluation of assisted oocyte activation, baseline clinical characteristics and ovarian stimulation protocols were intentionally standardized across study groups. Therefore, routinely reported background variables are not presented in detail. All clinical and laboratory procedures were performed by the same senior clinician (clinic director) and the same senior embryologist (laboratory director), each with over 10 years of experience.

The study population was divided into three groups:

- **Group I:**Conventional ICSI/Control Group (no activation applied), (n:50)
- **Group II:** ICSI + Piezoelectric Group (electrical activation applied), (n:50)
- **Group III:** ICSI + Calcium Ionophore Group (chemical activation applied), (n:50)

### Ethical considerations

This prospective non-randomized comparative study was approved by the Ethics Committee of Istanbul University, Istanbul Faculty of Medicine (approval number: E-29624016–050.99–322,082). This study was conducted in accordance with the principles of the Declaration of Helsinki. Written informed consent was obtained from all participants prior to their inclusion in the study.

The first study group (Group I) was enrolled initially. Subsequently, the faculty procured the calcium ionophore solution, and the third arm was treated. As the piezoelectric-assisted oocyte activation is part of the laboratory equipment, additional time was required for its procurement and installation. After the third group was treated, the piezoelectric-assisted oocyte activation became available, and the second arm was then treated. Therefore, this prospective non-randomized comparative study includes three groups that were treated sequentially in a prospective manner. As noted, the sample size calculation described in the Statistical Analysis section was based on fertilization rate, which was the primary endpoint of this study. The rationale for this choice was that the main objective was to evaluate the effectiveness of assisted oocyte activation techniques on oocyte activation and early embryological outcomes in patients with diminished ovarian reserve.

The three study groups were implemented sequentially rather than concurrently due to logistical constraints related to reagent procurement and the installation of the piezoelectric-assisted oocyte activation. Although the study was conducted prospectively, randomization was not feasible. Throughout the study period, no major changes were made to laboratory personnel, culture media, incubator systems, or ovarian stimulation protocols. This study was conducted over a 20-month period (October 2023–May 2025). Group I (Conventional ICSI) was applied between October 2023 and March 2024, Group III (Calcium ionophore) between April 2024 and October 2024, and Group II (Piezoelectric activation) between November 2024 and May 2025.

### Ovulation induction

All patients received either a gonadotrophin-releasing hormone (GnRH) antagonist protocol. Controlled ovarian hyperstimulation started on day 3 of the menstrual cycle using injectable gonadotropins (recombinant FSH, Gonal-F, EMD Serono, MA, USA) at a daily dose of 75–300 IU, depending on the patient’s age, antral follicle count, AMH level, body mass index, and previous response to ovarian stimulation. Follicular development and endometrial thickness were monitored through serial ultrasounds. Subcutaneous injections of cetrorelix 0.25 mg (Cetrotide 0.25 mg/day; Serono, Geneva, Switzerland) were initiated when the leading follicle reached 12–13 mm in diameter and continued until the day of human chorionic gonadotropin (hCG) administration. A dose of 6500 IU of hCG (Ovitrelle, Merck KGaA, Darmstadt, Germany) was administered to trigger follicular maturation when at least two follicles had reached a size of ≥ 17 mm.

### Oocyte pick-up (OPU) procedure

Transvaginal ultrasound-guided oocyte retrievals were performed 35–36 h after hCG administration. A Labotect device (Model 4014, Germany) and double-lumen OPU needles (Swemed 14,105, Sweden) were used for aspiration. Each follicle was aspirated individually into sterile tubes (Falcon 2003, USA/France) containing follicular fluid in ASP solution (Vitrolife, Sweden, EU) and GMOPS PLUS medium (Vitrolife, Sweden, EU), and examined under a stereomicroscope in the embryology laboratory (Olympus SZH10, Japan). The cumulus–oocyte complexes were further examined under an inverted microscope.Each follicle was aspirated individually to avoid cross-contamination between follicles and to ensure accurate identification and handling of the retrieved oocytes during the ICSI workflow. Although follicles were aspirated one by one, this did not necessarily require repeated puncture of the vaginal wall for every follicle, as multiple follicles could be accessed through the same puncture route whenever anatomically feasible. No analyses were performed on an individual follicle or follicular fluid basis.

Oocytes collected at the time of OPU were cultured after ICSI using a single-step culture system. Following ICSI, each oocyte/zygote was cultured individually in 20 µL microdrops of Total LP medium (CooperSurgical) under standard incubation conditions. Embryos were maintained in single culture throughout the culture period.

### Semen analysis and ICSI procedure protocol

Semen samples were obtained after 1–2 days of sexual abstinence. Sperm count and motility were assessed using phase-contrast microscopy with a Makler counting chamber, following the WHO 2021 guidelines [9].

Cumulus cells surrounding the oocytes were removed enzymatically using hyaluronidase and mechanically cleaned at least 2 h after oocyte retrieval (denudation of cumulus–oocyte complexes). The cleaned oocytes were then examined for maturation under an inverted microscope. Oocyte maturation and morphology was evaluated and classified as GV, MI, MII, or GIV. GIV (Grade IV) was defined as poor-quality oocytes characterized by severe morphological abnormalities. Intracytoplasmic sperm injection was performed under an inverted microscope (Nikon, Japan) equipped with a heated stage and an Integra micromanipulation system. All procedures were carried out in sterile ICSI dishes containing pre-warmed microdroplets of HEPES-buffered handling medium under mineral oil, maintained at 37 °C. Following enzymatic (hyaluronidase) and mechanical denudation, oocytes were assessed for nuclear maturity, and only metaphase II (MII) oocytes were selected for injection. Motile spermatozoa with normal morphology were selected from prepared semen samples and immobilized by gentle mechanical compression of the tail using the ICSI injection pipette in polyvinylpyrrolidone (PVP) prior to ICSI.Each oocyte was stabilized using a holding pipette, with the first polar body positioned at either the 6 or 12 o’clock position. A single immobilized spermatozoon was aspirated into the injection pipette and gently introduced through the zona pellucida and oolemma into the oocyte cytoplasm using controlled mechanical penetration. After injection, oocytes were immediately transferred into fresh culture medium and incubated under standard culture conditions (37 °C, 5–7% CO₂, and 5% O₂) until fertilization assessment. In the present study, ICSI was selected as the fertilization method for all patients despite the absence of male factor infertility. This approach was chosen due to the presence of diminished ovarian reserve and the expected retrieval of a very limited number of oocytes. In such cases, ICSI is commonly preferred to minimize the risk of total fertilization failure and to maximize fertilization efficiency when only one or a few oocytes are available. The use of ICSI in this context was therefore based on clinical judgment rather than male factor indications.

### Artificial oocyte activation (AOA) method

#### Calcium application

GM508 Cult-ActiveCalcium Ionophore, Germany is a ready-to-use activation solution that contains Ca^2^ ionophore and is prepared in GM501 Culture, a bicarbonate-buffered, ready-to-use medium containing gentamicin.

After ICSI of mature oocytes, calcium ionophore GM508 Cult-Active was prepared in 30 μL droplets in advance. After preparation, the drops were incubated for at least 4 h at 37 °C, pH 7.2–7.4, in a humidified atmosphere containing 5–7% CO₂ and 5% O₂. Immediately after ICSI, MII oocytes were transferred to a calcium ionophore-containing medium and incubated for 20 min. They were subsequently washed in a culture medium free of HEPES and MOPS and transferred into culture dishes. The oocytes were maintained in the incubator until fertilization assessment (Vitrolife, Sweden, EU). The 20-min exposure time was chosen in accordance with previous assisted oocyte activation protocols using calcium ionophore A23187, which indicate that 10–20 min of exposure is sufficient to achieve effective intracellular calcium elevation, especially in cases of suspected oocyte activation deficiency or poor oocyte quality [10]. We acknowledge that shorter exposure durations are also commonly used and that no universally standardized protocol exists; therefore, the selected duration represents a methodological choice.

#### Piezoelectric application

For piezoelectric-assisted oocyte activation, a stimulator unit, a culture dish, and an electroliquid solution (Zeus Bioscience) were used according to the manufacturer’s instructions. The electroliquid solution was pre-warmed in the incubator at 37 °C for approximately 20 min before ICSI, and 55–60 µL was used per patient.

Electrical stimulation was applied approximately 30 min after ICSI using predefined settings programmed in the device, which deliver standardized electrical pulses optimized for oocyte activation. As the stimulation parameters are automatically controlled by the device, no manual adjustment of voltage, pulse duration, or frequency was performed. After stimulation, oocytes were returned to culture dishes and maintained under standard incubation conditions.

The predefined settings consisted of standardized pulse intensity and duration as recommended by the manufacturer, ensuring reproducibility across all procedures. The stimulation was delivered using device-specific predefined settings, which consist of automated electrical pulse parameters, including pulse amplitude, duration (microsecond range), and frequency, designed to induce transient membrane depolarization and controlled intracellular calcium influx. These parameters are embedded within the system software and are not user-adjustable, thereby ensuring standardization and reproducibility across all procedures. Although exact numerical values of voltage and pulse characteristics are not accessible to the operator, this system operates within validated ranges established by the manufacturer and the prior experimental studies on electrical oocyte activation. This has now been clarified to allow a better comparison with other piezo-assisted activation approaches although direct parameter matching with manually adjustable systems may be limited.

#### Fertilization assessment

Fertilization was evaluated approximately 16–20 h after intracytoplasmic sperm injection (ICSI) using an inverted microscope equipped with Hoffman modulation contrast optics. Normal fertilization was defined by the presence of two distinct pronuclei (2PN) and two polar bodies (2 PB).

Abnormal fertilization patterns were also recorded, including:

- **0PN** (no pronuclear formation),
- **1PN** (single pronucleus),
- ** ≥ 3PN** (polypronuclear zygotes),
- **oocyte degeneration**.

Pronuclear morphology was assessed according to standard morphological criteria, including:

- size and symmetry of pronuclei,
- alignment and distribution of nucleolar precursor bodies (NPBs),
- cytoplasmic appearance.

However, zygote grading categories were not used for outcome comparisons, and fertilization outcomes were reported solely as fertilization rates based on 2PN formation. Only normally fertilized oocytes (2PN) were included in subsequent embryo culture and developmental analyses.

#### Embryo development

Embryo development was assessed at both the cleavage stage (day 2–3) and the blastocyst stage (day 5–6), depending on the individualized clinical transfer strategy. Cleavage-stage embryos were graded according to standard morphological criteria, including blastomere number, symmetry, and fragmentation, while blastocysts were evaluated based on expansion, inner cell mass, and trophectoderm quality.

To enable a unified comparison across patients with different transfer timings, embryos were subsequently classified into three overall developmental quality categories (good, moderate, poor) based on their stage-appropriate morphological grade, irrespective of the day of development. This day-independent classification was used for statistical analysis.

Embryo development was evaluated according to internationally accepted morphological criteria. Rather than presenting stage-specific definitions, embryo quality assessment followed standardized grading systems derived from the Istanbul consensus and the classification by Gardner DK [11, 12]. Cleavage-stage embryos were graded based on blastomere number, symmetry, and degree of fragmentation, while blastocyst-stage embryos were evaluated according to expansion stage, inner cell mass (ICM) quality, and trophectoderm morphology. To enable consistent comparison across patients with different embryo transfer timings, all embryos were subsequently categorized into three overall quality groups (good, moderate, poor) based on stage-appropriate morphology. This unified classification was used for statistical comparison; however, stage-specific embryo development data were also analyzed separately to minimize potential bias related to developmental timing.

### Embryo transfer procedure

#### Embryo transfer and assessment measures

Embryo transfers were performed on day 2, day 3, or day 5 under ultrasound guidance by the same infertility and reproductive endocrinology specialist experienced in IVF procedures. The day of embryo transfer (cleavage stage or blastocyst stage) was determined according to routine clinical practice and individual embryo development.

All embryo transfers were performed as fresh embryo transfer cycles. The number of embryos transferred (single or double embryo transfer) was determined based on patient age, embryo availability, and national regulations. Both first and repeated embryo transfer cycles were included in the study. Luteal phase support was provided with intramuscular progesterone in oil and vaginal progesterone suppositories. Surplus embryos were cryopreserved for subsequent cycles.

Reproductive outcomes were defined as follows:

- **Implantation:** Positive beta-HCG in serum 14 days after embryo transfer.
- **Clinical pregnancy:** A pregnancy defined by the presence of a gestational sac and fetal heartbeat on ultrasound.
- **Ongoing pregnancy:** A pregnancy that has progressed beyond 12 weeks of gestation with a viable fetus and a reduced risk of miscarriage.

### Statistical analysis

Statistical analysis was performed using SPSS software (version 25.0; IBM Corp., Armonk, NY, USA). The normality of continuous variables was assessed using Shapiro–Wilk test. As the data did not follow a normal distribution, non-parametric statistical methods were applied, and continuous variables were presented as median (minimum–maximum) values. Comparisons among the three study groups (Control, Piezoelectric activation, and Calcium ionophore activation) were performed using Kruskal–Wallis test. When a statistically significant difference was detected, pairwise comparisons were conducted using Dunn–Bonferroni post hoc test. Comparisons between two independent groups were performed using Mann–Whitney* U* test, where applicable. Categorical variables were compared using Pearson chi-square test. To evaluate the independent effect of assisted oocyte activation methods on fertilization rates, an age-adjusted multivariable logistic regression analysis was performed, and the results were reported as odds ratios (ORs) with 95% confidence intervals (CIs). A two-sided *p*-value < 0.05 was considered statistically significant. Based on effect sizes reported in previous studies by Fujii et al., Furuhashi et al., and Zander-Fox et al. [13–15], an a priori power analysis was performed using a significance level (α) of 0.05 and a power (1–β) of 80%. However, given the exploratory design of the study, a fixed sample size of 50 patients per group was included. Since only normozoospermic fresh sperm samples were included, a comparative analysis of sperm parameters between groups was not performed. To account for potential effects of ovarian stimulation protocols on embryological outcomes, ovarian stimulation protocol (GnRH antagonist) was included as a covariate in adjusted analysis. The day of embryo transfer was not included in the multivariable regression analysis, as transfer timing was determined based on clinical and embryological considerations and was comparable across groups.

## Results

**Fig. 1:**
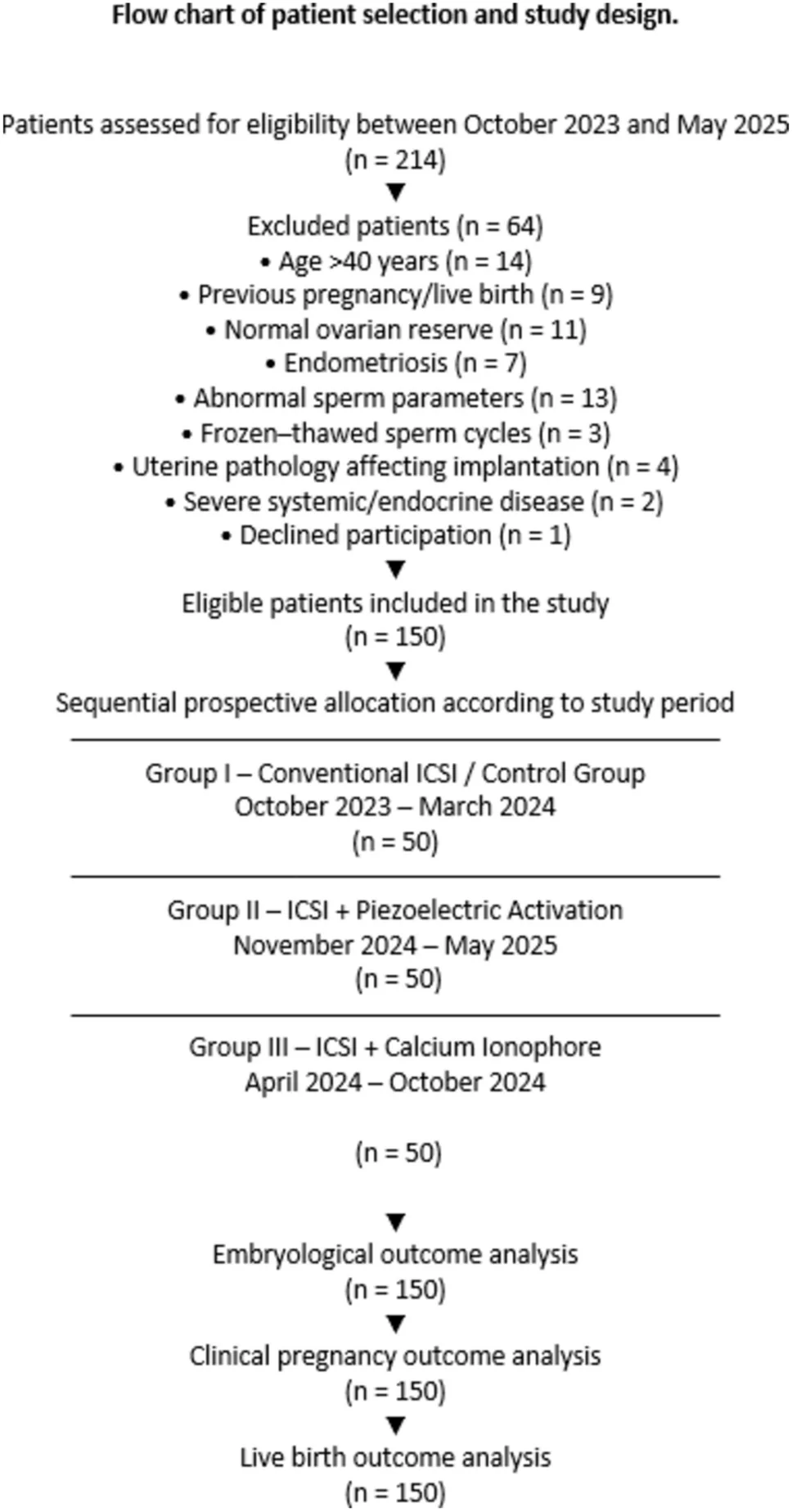
Flow chart demonstrating patient screening, exclusion process, sequential group allocation, and outcome analysis in the present prospective non-randomized comparative study. Patients were allocated sequentially according to the availability of assisted oocyte activation methods during the study period

A flow chart summarizing patient selection, exclusion criteria, sequential group allocation, and outcome assessment is presented in Fig. 1. Identical clinical and laboratory protocols were applied throughout the study period. Three groups were included in the study: Group I, the control group without artificial activation (*n* = 50); Group II, the piezoelectric activation group (*n* = 50); and Group III, the calcium ionophore activation group (*n* = 50). Demographic and clinical characteristics of all couples are summarized in Table 1. The mean age of the women was 38 years in the control group, 35 years in the piezoelectric group, and 38 years in the calcium ionophore group. A statistically significant difference in age was observed among the groups (*p* = 0.019). However, there was no significant difference between the control and calcium ionophore groups (*p* = 1.000), nor between the piezoelectric and calcium ionophore groups (*p* = 0.123). In the age-adjusted multivariable logistic regression analysis, neither the Piezo group (OR 2.10, 95% CI 0.36–12.37, *p* = 0.41) nor the calcium group (OR 0.64, 95% CI 0.17–2.43, *p* = 0.51) was independently associated with fertilization outcome (Table 2).

**Table 1:** Comparisons between groups according to demographic, hormonal, and embryological data. Data are presented as median (minimum–maximum) or number of patients (n) per group. The study included three groups: Control (Conventional ICSI, no artificial activation), Piezo (ICSI with Piezo electric activation), and Calcium (ICSI with calcium ionophore activation)

Parameters	Group I Conventional ICSI/ Control group * n*: 50	Group II ICSI + Piezo electric group* n*: 50	Group III ICSI + calcium Ionophore group * n*: 50	*p*-value	Pairwise comparisons *p*-value
	Median (Min–Max)	Median (Min–Max)	Median (Min–Max)		
Age	38 (27–46)	35 (21–43)	38 (23–44)	0.019*	Group I–Group II 0.021* Group I–Group III 1.000 Group II–Group III 0.123
Body mass index (BMI)	25 (19–40)	26.4 (19.5–36)	25.6 (18.4–39, 4)	0.843	
FSH	8.5 (2.95–20.95)	8.3 (4–25.1)	8.2 (0.3–22.2)	0.969	
LH	5.64 (2.2–18)	5.64 (2.7–19.5)	5.7 (0.34–19.5)	0.755	
E2	50.19 (14.4–151.3)	46 (23–187)	46 (5–218)	0.832	
PRL	20.6 (1.5–78)	14 (0.2–48.1)	15.7 (0.2–43)	0.051	
AMH	0.55 (0.07–1.05)	0.75 (0.08–1.09)	0.495 (0.08–1.08)	0.142	
Number of oocytes	4 (1–9)	3.5 (1–14)	3 (1–13)	0.964	
Metaphase II(MII)	3 (1–9)	2(0–13)	2.5(1–11)	0.478	
Injected MII oocytes	3 (1–9)	2 (0–13)	2.5 (1–11)	0.478	
2PN (fertilizedoocytes)	2 (0–7)	2 (0–13)	2.5 (0–11)	0.007	
Total fertilization failure (TFF), *n* (%)	8/50 (16%)	2/50 (4%)	3/50 (6%)	0.12	
Fertilization rate*	83 (0–100)	100 (0–100)	100 (0–100)	0.007*	Group I–Group II 0.007* Group I–Group III 1.000 Group II–Group III 0.071
Cleavage rate (%)	95 (50–100)	98 (80–100)	97 (70–100)	0.21	

Group I/ Q_1_–Q_3_: 50–100, Group II/ Q_1_–Q_3_:90–100, Group III/ Q_1_–Q_3_:57–100 Data are presented as median (minimum–maximum). Overall comparisons among groups were performed using Kruskal–Wallis test. Pairwise comparisons were conducted using Dunn–Bonferroni post hoc test when appropriate

**Table 2:** Age-adjusted multivariable logistic regression analysis for fertilization outcome

Variable	Odds ratio (OR)	95% confidence ınterval	*P* value
Group II vs. Group I	2.10	0.36–12.37	0.41
Group III vs. Group I	0.64	0.17–2.43	0.51
Age (per 1 year increase)	1.00	0.89–1.13	0.97

Age-adjusted multivariable logistic regression analysis. Results are presented as odds ratios (ORs) with 95% confidence intervals (CIs)

There were no statistically significant differences among the groups in baseline hormone levels (FSH, LH, E2, PRL, and AMH), total number of retrieved oocytes, number of mature (MII) oocytes, and Body Mass Index (BMI). A statistically significant difference was observed in fertilization rates among the groups (*p* = 0.007). Pairwise comparisons revealed a significant difference between the piezoelectric activation group and the control group (*p* = 0.007), favoring the piezoelectric group. Although the calcium ionophore group exhibited a higher fertilization rate compared to the control group, this difference did not reach statistical significance (Table 1). No abnormal fertilization events (1PN or 3PN) were observed in any of the study groups.

No statistically significant differences were observed among the groups in the number of Grade I embryos (*p* = 0.172) or Grade II embryos (*p* = 0.687). However, a statistically significant difference was found in the number of Grade III embryos (*p* = 0.011) (Table 2). Pairwise analysis revealed a significant difference between the control group and the piezoelectric activation group, favoring the piezoelectric group, which exhibited fewer poor-quality (Grade III) embryos (Table 3).

**Table 3:** Overall embryo developmental quality independent of developmental stage. Data are presented as number of embryos and median (min–max). Embryos were classified as good-quality (GI), average-quality (GII), or poor-quality (GIII)

Embryo grade	Group I Conventional ICSI Control Group * n*: 50		Group II ICSI + Piezoelectric Group * n*: 50		Group III ICSI + Calcium Ionophore Group * n*: 50		*p*-value
	Number of embryos	Median (Min–Max)	Number of embryos	Median (Min–Max)	Number of embryos	Median (Min–Max)	
Good-quality embryos (GI)	57	1 (0–6)	79	1 (0–8)	69	1 (0–5)	0.172
Average-quality embryos (GII)	35	0 (0–3)	42	1 (0–4)	32	0 (0–3)	0.687
Poor-quality embryos (GIII)	30	0 (0–6)	9	0 (0–3)	11	0 (0–2)	0.011*

Study groups were defined as follows: Control (conventional ICSI), Piezo (ICSI + piezoelectricactivation), and Calcium (ICSI + calcium ionophore activation). Embryo quality was initially assessed according to the appropriate developmental stage (cleavage-stage or blastocyst-stage) using internationally accepted criteria

For comparative analysis, embryos were subsequently categorized into overall developmental quality groups independent of the day of culture. No Grade IV embryos were observed in the study population. *P*-values represent between-group comparisons for each embryo quality category using Kruskal–Wallis test. A statistically significant difference was observed only for poor-quality embryos (GIII)

Stage-specific embryo development was additionally evaluated to address the potential limitation of day-independent embryo quality classification. Delayed cleavage embryos (≤ 4-cell embryos on day 3) and embryos remaining at the morula stage on day 5 were numerically more frequent in the control group, whereas the piezoelectric and calcium ionophore groups showed higher proportions of stage-appropriate embryos. These findings suggest that assisted oocyte activation may be associated not only with fertilization outcomes but also with improved developmental progression (Table 4).

**Table 4:** Stage-specific embryo developmental kinetics according to the day of embryological assessment

Developmental parameter	Group I Conventional ICSI Control group * n*: 50	Group II ICSI + piezoelectric group * n*: 50	Group III ICSI + calcium ıonophore group * n*: 50	*p*-value
Day 3 embryos				
≤ 4-cell embryos, *n* (%)	18/82 (22.0%)	7/86 (8.1%)	9/75 (12.0%)	0.041
≥ 6-cell embryos, *n* (%)	64/82 (78.0%)	79/86 (91.9%)	66/75 (88.0%)	0.041
Day 5 embryos				
Morula-stage embryos, *n* (%)	10/40 (25.0%)	4/44 (9.1%)	5/37 (13.5%)	0.038
Blastocyst-stage embryos, *n* (%)	30/40 (75.0%)	40/44 (90.9%)	32/37 (86.5%)	0.038

Data are presented as number of embryos and percentages within each developmental stage category. Embryo developmental progression was evaluated separately on day 3 and day 5 to assess potential differences in developmental kinetics among the study groups

No significant differences were observed among groups in cleavage rates or total fertilization failure rates. When implantation rates were evaluated, the highest rate was observed in the calcium ionophore group (37%); however, no statistically significant differences were found among the groups (*p* = 0.224) (Table 5). Similarly, there were no significant differences among the groups in clinical pregnancy, ongoing pregnancy, or live birth rates. All live-born infants were clinically healthy, with no congenital anomalies or known genetic disorders detected at birth.

**Table 5:** Pregnancy outcomes among the study groups. Data are presented as number of pregnancies/number of embryo transfers (%). Groups: control (Conventional ICSI), Piezo (ICSI + Piezo electric activation), calcium (ICSI + calciumionophoreactivation)

Pregnancy outcome	Group I Conventional ICSI Control group * n*: 50	Group II ICSI + Piezoelectric group * n*: 50	Group III ICSI + Calcium ıonophore group * n*: 50	*p*-value
Implantation rate	7/36 (% 19)	10/28 (% 36)	11/30 (% 37)	0.224
Clinic pregnancy rate	6/36 (% 17)	8/28 (% 29)	10/30 (% 33)	0.363
Ongoing pregnancy	5/36 (% 14)	4/28 (%14)	7/30 (% 23)	0.590
Live birth rate	5/36 (% 14)	4/28 (% 14)	7/30 (% 23)	0.538
Day 2 transfer, *n* (%)	10/36 (27.8%)	8/28 (28.6%)	9/30 (30.0%)	0.95
Day 3 transfer, *n* (%)	16/36 (44.4%)	12/28 (42.9%)	13/30 (43.3%)	0.98
Day 5 transfer, *n* (%)	10/36 (27.8%)	8/28 (28.6%)	8/30 (26.7%)	0.97

Pregnancy outcomes were compared between groups using Pearson chi-square test. Embryo transfer day distribution was compared using Pearson chi-square test

## Discussion

This study aimed to investigate whether artificial oocyte activation improves fertilization rates and pregnancy outcomes in ICSI cycles in couples with diminished ovarian reserve. This study demonstrated a statistically significant difference in fertilization rates among the groups, with both intervention groups showing higher fertilization rates compared to the control group. Although pregnancy rates were higher in both the calcium ionophore and piezoelectric activation groups compared to the control group, the differences were not statistically significant.

When interpreted within the framework of the Poseidon classification, the majority of patients included in this study would correspond to Poseidon Groups 3 and 4, characterized by diminished ovarian reserve and an expected poor response to ovarian stimulation [16]. This classification further supports the clinical relevance of evaluating assisted oocyte activation strategies in this challenging patient population. In women with diminished ovarian reserve, repeated IVF attempts are frequently required due to the limited number of oocytes obtained per cycle. Each unsuccessful cycle imposes a substantial psychological, physical, and financial burden on patients, particularly in poor responders with low cumulative chances of success. In this context, the exploratory use of assisted oocyte activation may be considered as a strategy aimed at maximizing fertilization potential when only a very limited number of oocytes are available rather than as a routine intervention. Although current guidelines restrict the indication for AOA to specific clinical scenarios, its careful application in highly selected poor responders may represent an attempt to enhance the probability of fertilization and embryo development within a single IVF cycle, thereby potentially reducing the need for repeated treatment cycles. To address the potential confounding effect of maternal age, we performed an age-adjusted multivariable logistic regression analysis. After adjustment for age, neither the Piezo-assisted nor the calcium activation group was independently associated with fertilization rates compared with the control group. In addition, age was not a significant predictor in the model. These findings indicate that the fertilization rates observed in this study were not influenced by age-related differences between groups and support the robustness of the primary results. The consistency between unadjusted and age-adjusted analyses suggests that the choice of oocyte activation technique did not have a clinically meaningful impact on fertilization success in the studied population. Therefore, the findings should be considered exploratory and hypothesis-generating rather than definitive evidence of treatment efficacy.

Specifically, the fertilization rate in the piezoelectric activation group was significantly higher compared to the control group. This finding is consistent with previous studies [17]. Several previous studies have investigated the role of assisted oocyte activation in patients with poor or failed fertilization, employing various activation agents including calcium ionophores, electrical stimuli, and mechanical methods. Early reports on chemical AOA, particularly using calcium ionophores, such as ionomycin and A23187, demonstrated increased fertilization rates in couples with a history of low fertilization after conventional ICSI, suggesting that calcium-mediated oocyte activation plays a central role in overcoming oocyte activation deficiency [18]. However, these improvements were not consistently associated with enhanced embryo quality or clinical pregnancy outcomes across all studies, particularly in populations without well-defined activation defects. In contrast, electrical activation techniques, such as piezoelectric pulses applied post-ICSI, have been hypothesized to more closely mimic physiological calcium oscillations within the oocyte, leading to more effective downstream signaling and cytoskeletal reorganization necessary for normal fertilization [19]. A prospective randomized study by Aytac et al. [20] demonstrated that calcium ionophore-based assisted oocyte activation significantly improved fertilization rates; however, similar to our findings, this improvement did not consistently translate into higher clinical pregnancy or live birth rates. These findings support the concept that while artificial activation may overcome initial fertilization deficiencies, downstream developmental competence may remain unaffected. Recent comparative analysis has suggested that although electrical AOA may result in higher fertilization rates than certain chemical agents in selected subgroups, the translation into improved blastocyst formation, implantation, and live birth outcomes remains inconsistent. For example, Mansour et al., 2009 reported a significant increase in fertilization with piezoelectric activation compared to calcium ionophore, but no difference in clinical pregnancy rates, a finding that aligns with the current data [21].

Deficiencies in PLCζ expression or function have been associated with fertilization failure, increased sperm DNA fragmentation, and impaired chromatin decondensation. While artificial oocyte activation methods such as calcium ionophores induce a single transient calcium rise, physiological fertilization involves repetitive calcium oscillations driven by PLCζ. Recent evidence has further clarified the central role of PLCζ in fertilization competence. In addition, a 2025 study by Soukaina Azilet al. and colleagues demonstrated that reduced PLCζ expression is strongly associated with increased sperm DNA fragmentation and defective chromatin decondensation, suggesting that impaired sperm-derived activation signals may affect not only fertilization but also embryo developmental competence [22]. More recently, a comprehensive 2022 review on sperm–oocyte interactions has further reinforced that PLCζ-driven calcium oscillations represent the physiological gold standard for successful oocyte activation, integrating molecular, cellular, and clinical evidence across mammalian species [23]. In this context, assisted oocyte activation should be interpreted as a compensatory intervention rather than a physiological substitute. Electrical or piezoelectric activation may partially mimic this process by inducing more dynamic calcium influx, although it still does not fully replicate the endogenous oscillatory pattern. This fundamental difference in calcium signaling dynamics may explain why AOA improves fertilization rates without consistently enhancing implantation, pregnancy, or live birth outcomes. Indeed, while AOA may overcome initial activation failure related to PLCζ deficiency, it does not address underlying defects in oocyte quality, mitochondrial function, or genomic integrity, which are particularly relevant in patients with diminished ovarian reserve. An important consideration is that the present study intentionally excluded patients with severe male factor infertility or abnormal sperm parameters, including those most likely to exhibit sperm-related oocyte activation deficiency associated with impaired PLCζ signaling. Therefore, the study population does not represent the classical indication group for assisted oocyte activation. This design was chosen to isolate the potential contribution of oocyte-related factors in patients with diminished ovarian reserve while minimizing confounding effects of sperm dysfunction. However, the exclusion of patients with proven activation deficiency may partly explain the limited impact of AOA on downstream clinical outcomes despite improved fertilization rates.

With the observed increase in fertilization rates, piezoelectric activation may potentially increase the number of transferable embryos in patients with diminished ovarian reserve. However, this observation should be interpreted cautiously given the exploratory nature of the study and the absence of significant differences in major clinical outcomes.The proportions of good, moderate, and poor-quality embryos were compared among the three groups. No statistically significant differences were observed in the rates of good and moderate-quality embryos; however, a higher proportion of poor-quality embryos was found in the control group without artificial oocyte activation. This finding is consistent with our hypothesis and may suggest a potential benefit of assisted oocyte activation with respect to embryo quality; however, this observation should be interpreted cautiously.The increased rate of poor-quality embryos in the absence of artificial activation may be attributed to insufficient oocyte activation and subsequent impairment of early embryonic development. These results underscore the importance of activation techniques in optimizing embryological outcomes and suggest that artificial oocyte activation could help reduce the incidence of poor-quality embryos.In a prospective cohort study conducted in 2023 involving 120 patients with poor ovarian response undergoing IVF/ICSI, the group receiving artificial oocyte activation demonstrated increased fertilization rates (89%), embryo development rates (96%), and an average of 1.52 Grade A embryos. In the control group, these values were 83.04%, 92.55%, and 1.04, respectively. However, no statistically significant difference was observed in clinical pregnancy rates between the groups, which were approximately 34% [24]. Similarly, a study by Tsaiet al. demonstrated that the application of calcium ionophore during IVF–ICSI cycles in patients over 40 years of age with diminished ovarian reserve may improve embryo quality [25]. While a reduction in the proportion of poor-quality embryos was observed with artificial oocyte activation, these embryological improvements did not result in superior clinical outcomes. Therefore, embryo morphology alone may not fully reflect implantation potential or chromosomal competence in this patient population.

In contrast to the study by Ebner et al., the present study observed the highest pregnancy rate in the calcium ionophore group (36.7%) when comparing pregnancy outcomes among the groups [26]. Artificial oocyte activation thus appeared to have a positive effect on pregnancy rates, although this effect did not reach statistical significance. No statistically significant differences were observed in clinical pregnancy, ongoing pregnancy, or live birth rates. Therefore, while AOA can enhance the number of fertilized oocytes, its impact on ultimate pregnancy outcomes may be limited by these additional variables.Although the highest biochemical and clinical pregnancy rates were observed in the calcium ionophore group, these differences did not reach statistical significance. This finding suggests that while chemical activation may show a trend toward improved clinical outcomes, the current sample size may be insufficient to detect meaningful differences.

In this study, although a statistically significant difference in fertilization rates was observed between the control group and the piezoelectric activation group (Group II), no significant difference was found between the control group and the calcium ionophore activation group (Group III). No statistically significant difference was observed between the two assisted oocyte activation methods, suggesting that both chemical and electrical activation may provide comparable effects on fertilization outcomes. However, given the sequential non-randomized design and limited sample size, these findings should be interpreted cautiously and considered exploratory.This study contributes to the growing body of evidence regarding assisted oocyte activation techniques in patients with diminished ovarian reserve. However, several limitations should be considered. As the findings are derived from a specific patient population, caution is warranted when generalizing the results. Since all groups consisted of infertile individuals, the outcomes cannot be directly compared to the physiological responses observed in fertile women.

This study has several limitations that should be considered. First, it was conducted at a single center, which may limit the generalizability of the findings to other clinical settings with different patient populations and laboratory practices. In addition, the non-randomized, sequential allocation of study groups introduces the possibility of temporal confounding as changes over time in laboratory conditions or patient characteristics may have influenced the results. Furthermore, fertilization rate, although selected as the primary endpoint, represents an intermediate outcome and may not necessarily predict ultimate clinical success, particularly in women with diminished ovarian reserve. Another limitation is the omission of detailed baseline and stimulation-related variables; although these parameters were standardized across groups, subtle differences may still have influenced the outcomes. Similarly, while major uterine pathologies were excluded, variations in endometrial receptivity cannot be fully ruled out. The absence of preimplantation genetic testing also limits the ability to directly assess embryo aneuploidy risk.Although no congenital anomalies were observed among live-born infants, the potential epigenetic and long-term developmental effects of assisted oocyte activation cannot be excluded and should be interpreted with caution in accordance with current ESHRE recommendations [27]. While previous studies have reported reassuring neonatal outcomes and no significant increase in aneuploidy rates [28, 29], long-term safety remains an area requiring further investigation.Finally, the exclusion of frozen–thawed sperm cycles may limit the applicability of the findings as sperm cryopreservation has been associated with impaired oocyte activation capacity. In addition, the use of a day-independent embryo quality classification, although facilitating comparison across patients, may have obscured differences in developmental kinetics and embryo viability.

## Conclusion

To conclude, assisted oocyte activation was associated with numerically higher fertilization rates in this cohort of patients with diminished ovarian reserve; however, this improvement did not translate into significant differences in implantation, pregnancy, or live birth outcomes. These findings suggest that while assisted oocyte activation may improve early embryological outcomes, its impact on clinically meaningful reproductive outcomes remains uncertain. Given the prospective sequential non-randomized design, the absence of significant differences in adjusted analysis, and the lack of improvement in major clinical outcomes, these findings should be interpreted with caution. The results should be considered exploratory and hypothesis-generating rather than definitive evidence of clinical benefit. Further adequately powered randomized controlled trials are required to determine whether assisted oocyte activation provides meaningful improvements in reproductive outcomes before its routine use in patients with diminished ovarian reserve can be recommended.

## Data Availability

The data that support the findings of this study are not publicly available due to restrictions on participant privacy but are available from the corresponding author upon reasonable request.

## Declarations

### Registry and the registration no. of the study/trial

N/A.

### Animal studies

N/A.
